# TRIP13 overexpression in hepatocellular carcinoma: implications for poor prognosis and immune cell infiltration

**DOI:** 10.1007/s12672-023-00792-2

**Published:** 2023-09-22

**Authors:** Jiapeng Xue, Hongfen Wu, Yun Shi, Zhi Li

**Affiliations:** 1 Department of General Surgery, Hubei Clinical Research Center for Precise Diagnosis and Treatment of Liver Cancer, Taihe Hospital, Hubei University of Medicine, Shiyan, China; 2 Department of Gastroenterology, West China (Sanya) Hospital, Sichuan University, Sanya, China; 3 Interventional Cancer Institute of Chinese Integrative Medicine, Putuo Hospital, Shanghai University of Traditional Chinese Medicine, Shanghai, China; 4 Hubei Provincial Clinical Research Center for Umbilical Cord Blood Hematopoietic Stem Cells, Taihe Hospital, Hubei University of Medicine, Shiyan, China

**Keywords:** Thyroid hormone receptor interactor 13, Hepatocellular carcinoma, Prognosis, The Cancer Genome Atlas, Nomogram, Immune cell infiltration

## Abstract

**Purpose:**

The overexpression of TRIP13 has been observed in many types of cancer and has been identified as an oncogene. However, its role in hepatocellular carcinoma (HCC) has not been extensively studied. This study aimed to investigate the expression of TRIP13 in HCC and its impact on immune cell infiltration and prognosis.

**Methods:**

We analyzed TCGA and GSE62232 datasets to assess TRIP13 expression in HCC. Kaplan–Meier and subgroup analysis were performed to examine the correlation between TRIP13 expression and HCC. Univariate and Cox regression analysis were conducted to determine the predictive value of TRIP13 in assessing patient outcomes. A nomogram was developed using TRIP13 mRNA expression to predict HCC prognosis. TRIP13 expression was validated using immunohistochemistry in our patient cohort. Survival and subgroup analyses were conducted to investigate the role of TRIP13 in HCC prognosis.

**Results:**

The results indicated that TRIP13 upregulation in HCC was a strong independent predictor of poor outcome, as determined by Kaplan–Meier and Cox regression analyses. A high AUC value of 0.982 from ROC curves suggested that TRIP13 upregulation could serve as a reliable diagnostic indicator for HCC. The immunohistochemical validation of TRIP13 expression in the patient cohort confirmed its prognostic significance, and high TRIP13 expression was found to be associated with increased infiltration of Th2 cells and decreased infiltration of neutrophils, Th17 cells, and dendritic cells.

**Conclusion:**

These findings suggest that TRIP13 could be a potential prognostic biomarker for HCC.

**Supplementary Information:**

The online version contains supplementary material available at 10.1007/s12672-023-00792-2.

## Introduction

Hepatocellular carcinoma (HCC) is one of the most common malignancies in the digestive system, with the sixth highest incidence of all cancers and the second highest mortality [1]. It mainly affects people in Africa and Asia, particularly in China where it is most prevalent [2]. While surgical resection and liver transplantation are the most effective treatment, many HCC patients are diagnosed at a late stage when these options are no longer feasible, resulting in a low 5-year survival rate of only around 25% [3]. Moreover, even after successful surgery, the chances of HCC recurrence are high [4]. Despite the identification of several biomarkers associated with HCC, the available treatment options are still inadequate, and reliable prognostic markers are lacking [5]. Therefore, identifying new molecular biomarkers associated with HCC could contribute to the early detection and treatment of this disease.

In 1995, it was discovered that the TRIP13 protein, which interacts with the thyroid receptor in breast cancer cell lines, belongs to the AAA + ATPase superfamily [6]. The TRIP13 protein consists of 432 amino acids and plays a crucial role in gene transcription, aiding in the binding of thyroid hormones to thyroid hormone receptors [7]. TRIP13 is known to regulate meiotic recombination, spindle assembly checkpoints, and chromosomal synaptic connections [8]. Despite its importance in meiosis, it has been shown to be overexpressed or elevated in many types of cancer, such as those affecting the colon, head and neck, and prostate. It modulates tumor cell growth, lifespan, and invasiveness in these cancers [9]. Although TRIP13 expression has been observed to be elevated in HCC, its clinical significance and role in the disease remain poorly understood [10].

The tumor microenvironment is a crucial factor in tumorigenesis, progression, metastasis, and therapeutic response [11]. An important component of the tumor microenvironment is tumor-infiltrating immune cells, which promote tumorigenesis and progression in various ways [12]. Regulatory T cells (Tregs), dendritic cells, T cells, and natural killer cells (NK) are some of the immune-infiltrating cells that have been associated with unfavorable clinical outcomes and can be utilized for evaluating clinical outcomes and drug response in cancer patients [13]. While immunotherapy is an important approach in tumor treatment, the immune microenvironment can reduce the effectiveness of immunotherapy by promoting immune tolerance and evasion [14]. Therefore, it is essential to investigate the correlation between tumor-related genes and immune cell infiltration, given the important role that immune cells play in tumor prognosis and treatment. This study investigates the function of TRIP13 in human HCC as well as its function in immune cell infiltration.

## Materials and methods

### Data mining on the TCGA-HCC and GSE62232 datasets

This study employed 374 primary HCC tissues and 50 normal control tissues obtained from the TCGA data repository (https://genome-cancer.ucsc.edu/). The Gene Expression Omnibus (GEO) database (https://www.ncbi.nlm.nih.gov/geo/) GSE62232 was utilized to confirm differential expression of TRIP13 between the two tissue types. To determine TRIP13 mRNA levels in HCC tissues, we utilized the R packages edgeR and ggplot2 to normalize, label, and compare raw count data with those of normal controls. Table S1 presents additional characteristics of the TCGA participants included in this analysis. Patients selected for this study met the following criteria: (1) a pathology-based diagnosis of HCC, and (2) comprehensive information on survival outcomes.

### Development and assessment of a prognostic nomogram based on TRIP13

We conducted an investigation into the impact of TRIP13 on the prognosis of HCC patients and validated our findings using additional data sources. In order to assess the diagnostic performance of TRIP13 for HCC, we generated a receiver operating characteristic curve and calculated the corresponding area under the curve. Our aim was to identify independent prognostic factors for HCC through univariate and multifactorial analyses, and subsequently construct prognostic nomograms based on these factors. We evaluated the degree of calibration by constructing a calibration curve.

### Patients in an independent validation cohort

A study was conducted on 90 patients who underwent resection surgery for liver cancer at West China (Sanya) Hospital, Sichuan University, between January 2011 and February 2017. Imaging and histopathological examinations were conducted after surgery to confirm the diagnosis of primary liver cancer, and only patients with complete clinicopathological data were included. Patients who had received radiotherapy, chemotherapy, or interventional therapy were excluded (Table 1).

**Table 1:** Association of TRIP13 levels with clinicopathological parameters of HCC patients

Parameters	TRIP13 protein levels		*χ*^2^	P value
	Low (n = 38)	High (n = 52)		
Gender			0.698	0.403
Male	31	39		
Female	7	13		
Age (year)			0.212	0.645
≤ 60	28	36		
> 60	10	16		
TNM stage			4.555	0.033
I	24	21		
II	14	31		
AFP (ng/mL)			5.947	0.015
≤ 50	10	27		
> 50	28	25		
Tumor size (cm)			1.064	0.302
≤ 5	20	33		
> 5	18	19		
Tumor number			0.0	0.991
Solitary	30	41		
Multiple	8	11		
HBsAg			0.206	0.650
Negative	9	10		
Positive	29	42		
HCvAb			0.050	0.822
Negative	37	51		
Positive	1	1		
Encapsulation			6.397	0.011
Incomplete	11	29		
Complete	27	23		
Tumor differentiation			0.171	0.679
I–II	25	32		
III–IV	13	20		

### Immunohistochemistry study

The tissue microarray was made using described methods and steps [15]. The specifics of Immunohistochemistry have been explained [16]. Already-made TMAs were used for TRIP 13 immunohistochemistry. We used xylene to remove wax, alcohol to rehydrate, and 3% hydrogen peroxide to stop endogenous peroxidases. Next, slides were incubated with a 1:500 dilution of rabbit anti-human TRIP13 monoclonal antibody (Abcam, MA, USA). After washing in PBS, incubating with secondary antibody for 30 min, and developing with PBS, sections were developed with diaminobenzidine solution (Beyotime, Shanghai, China). Weakly positive was worth one point, moderately positive was worth two, and strongly positive was worth three. We scored positive cells as follows: 0 for 10%, 1 for 10–25%, 2 for 50–70%, and 3 for 100%. Multiplying staining by stained cells yielded protein level.

### Immunocell infiltration analysis

To explore the potential relation between TRIP13 expression and infiltration of immune cell, we employed the Gene Set Enrichment Analysis (GSEA) package to analyze the TCGA cohort. We evaluated the correlation between TRIP13 expression and the level of infiltration caused by 24 immune cells, and then narrowed our focus to the top four cell types that demonstrated the strongest association. Subsequently, we conducted a comprehensive investigation of these four immune cell types.

### Statistical analysis

We conducted statistical analysis using several software programs, including SPSS 22.0 (IBM, Armonk, NY, USA), R (3.6.3), and GraphPad Prism 8.0 (GraphPad Software, La Jolla, CA, USA). Based on the median level of TRIP13 expression, we divided the patients into high and low expression groups. A *P*-value of less than 0.05 was considered statistically significant.

## Results

### Overexpression of TRIP13 in HCC compared to normal tissues

**Fig. 1:**
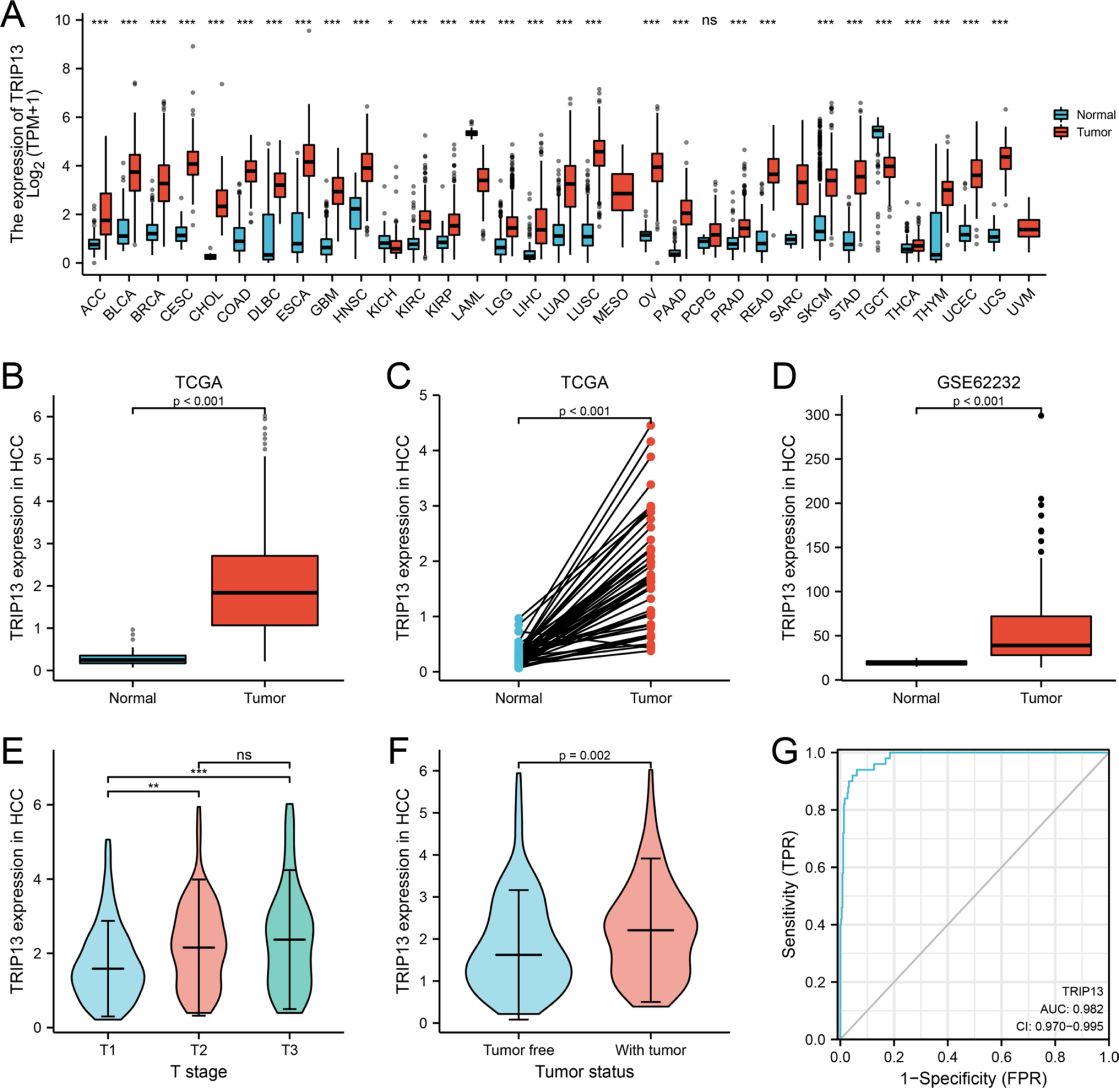
A study of TRIP13 expression levels in HCC and other types of cancers among the TCGA dataset. **A** The expression levels of TRIP13 in various types of tumors from TCGA database. **B** Detection of TRIP13 expression in HCC (n = 374) and normal tissue (n = 50). **C** TRIP13 expression in HCC (n = 50) and adjacent tissues (n = 50). **D** TRIP13 expression levels in HCC (n = 81) and normal tissue (n = 10) in GSE62232 database. **E** The relationship between TRIP13 expression and T stage in HCC (n = 358). **F** HCC tumor status and TRIP13 expression (n = 355); **G** A ROC analysis of TRIP13 in HCC (n = 424)

Analysis of the TCGA database revealed that the majority of human tumors overexpress TRIP13 mRNA relative to normal tissues (Fig. 1A). Notably, the expression of TRIP13 mRNA was significantly higher in tumor tissues (n = 374) than in normal liver tissue (n = 50) and paratumoral tissue (n = 50) (*P < *0.001) (Fig. 1B, C). The GSE62232 dataset further revealed a distinct expression pattern of TRIP13 between HCC (n = 81) and normal tissue (n = 10) (Fig. 1D). Moreover, a strong correlation between the level of TRIP13 mRNA expression and the tumor’s T stage (Fig. 1E) as well as the tumor status (Fig. 1F) was observed. Moreover, the ROC curves suggested that TRIP13 upregulation had high diagnostic value for HCC (AUC = 0.982) (Fig. 1G and Table S1). The TRIP13 mRNA expression level was found to be strongly associated with various clinicopathological features, including the T stage, AFP level, vascular invasion, tumor status, overall survival, and progression-free interval (PFI) events (all P < 0.05), but not with other clinicopathological factors.

### TRIP13 overexpression predicts poor prognosis in HCC

**Fig. 2:**
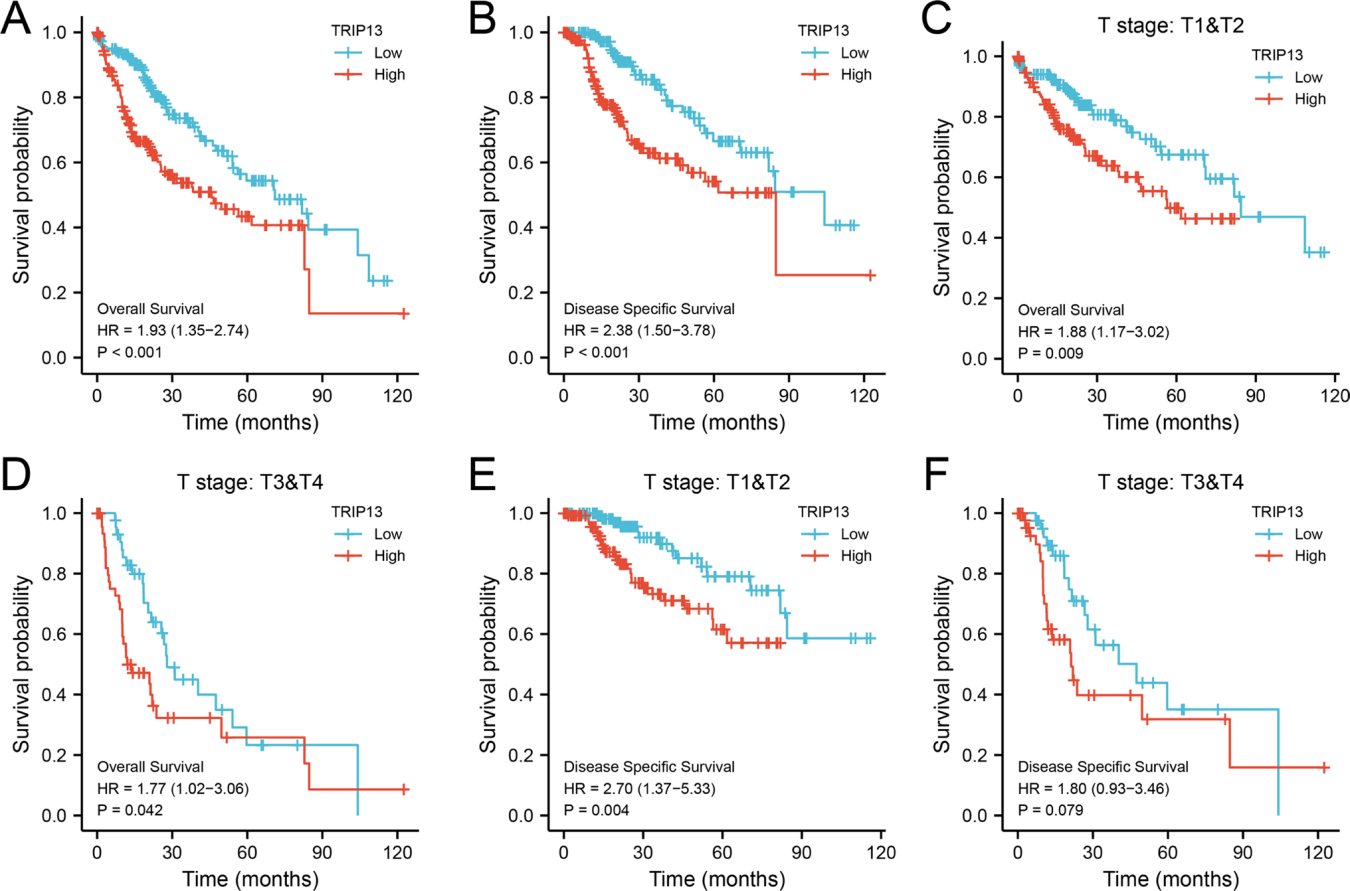
TRIP13’s prognostic value in HCC based on TCGA databset. **A** Overall survival curves based on Kaplan–Meier analysis (n = 373). **B** Disease specific survival (DSS) based on Kaplan-Meier analysis (n = 365). **C**, **D** Analysis of OS subgroups based on T stage. **E**, **F** DSS subgroup analyses based on T stage

The overall survival (OS) and disease-specific survival (DSS) survival curves indicated that patients with high TRIP13 had significantly shorter OS (HR = 1.93, 95% CI (1.35, 2.74), *P < *0.001) and DSS (HR = 2.38, 95% CI (1.50, 3.78), *P *< 0.001) than those with low TRIP13 (Fig. 2A, B). TRIP13 expression, T stage, M stage, and OS in HCC, as well as Child–Pugh grade and DSS in HCC, were all found to be significantly associated (all *P *< 0.05). In a multivariate analysis, high TRIP13 expression emerged as an independent negative predictor of both OS (HR = 2.023, 95% CI (1.282–3.193), *P* = 0.002) and DSS (HR = 6.376, 95% CI (2.325–17.486), *P* = 0.001) in HCC patients (Tables S2, S3). Subgroup analysis using the TNM stage revealed that it was an independent indicator for both OS and DSS (*P *< 0.05). Importantly, high expression of TRIP13 was related to adverse outcomes in HCC patients regardless of TNM stage, as evidenced by our results (Fig. 2C–F).

### Prediction of HCC prognosis based on a nomogram based on TRIP13

**Fig. 3:**
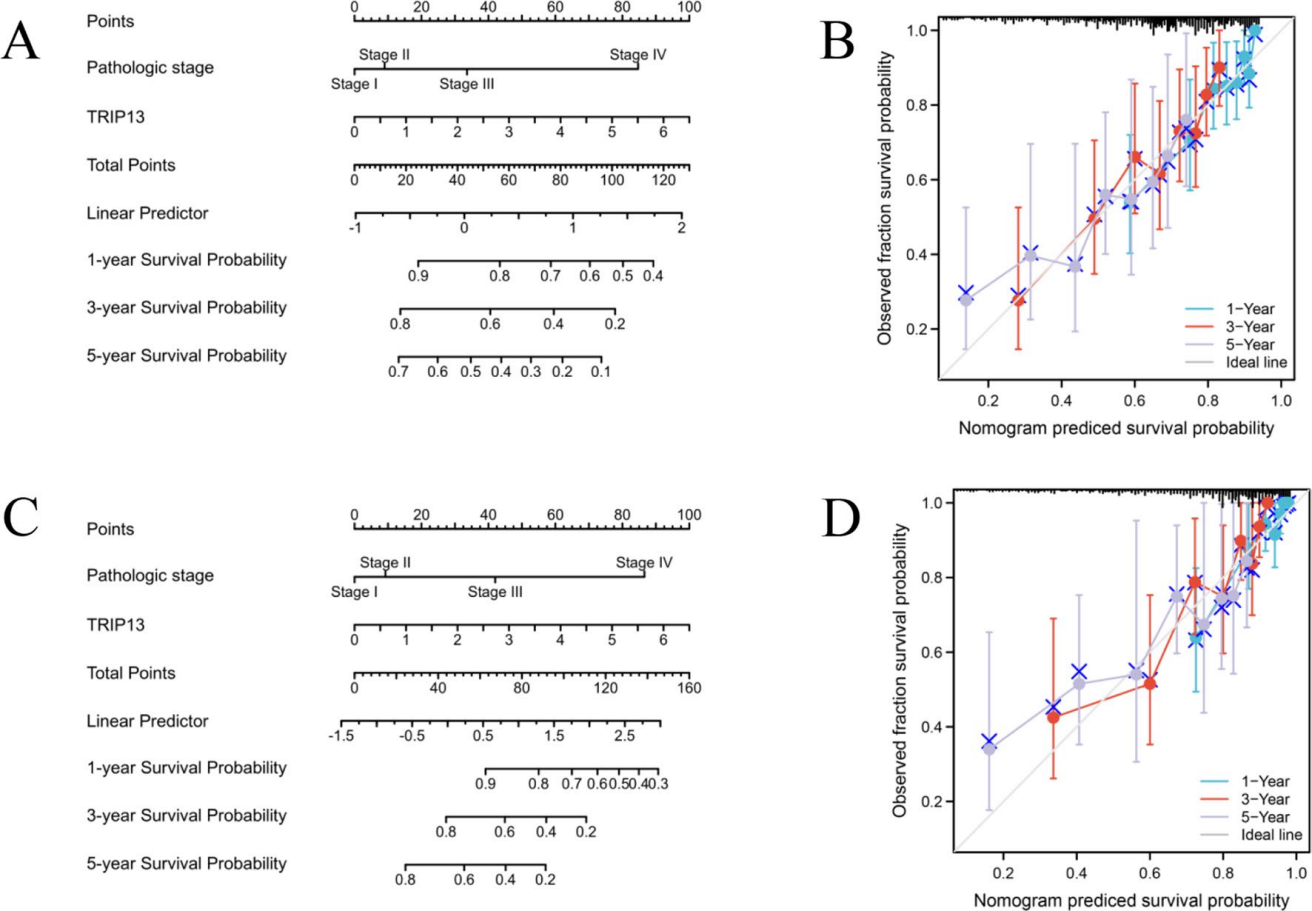
Prognostic nomograms derived from TCGA data for patients with HCC. **A** An integrated prognostic nomogram for overall survival (OS) of HCC that includes TRIP13 and other prognostic factors. **B** Predictive accuracy of the nomogram for OS based on its calibration curve. **C** An integrated prognostic nomogram for disease specific survival (DSS) of HCC that includes TRIP13 and other prognostic factors. **D** Predictive accuracy of the nomogram for DSS based on its calibration curve

The prognostic value of TRIP13 in HCC patients was verified by constructing a nomogram based on TRIP13 mRNA expression and pathologic stage. The calibration plots showed a significant correlation between the predicted probability of fraction survival (OS and DSS) after hepatectomy and the observed 1-, 3-, and 5-year survival rates. The genomic-clinicopathologic nomogram demonstrated a C-index of 0.677 (95% CI 0.650–0.704) for OS and a C-index of 0.767 (95% CI 0.737–0.796) for DSS (Fig. 3), thereby further confirming the prognostic value of TRIP13 in HCC.

### Validation TRIP13 expression in HCC using an independent cohort

**Fig. 4:**
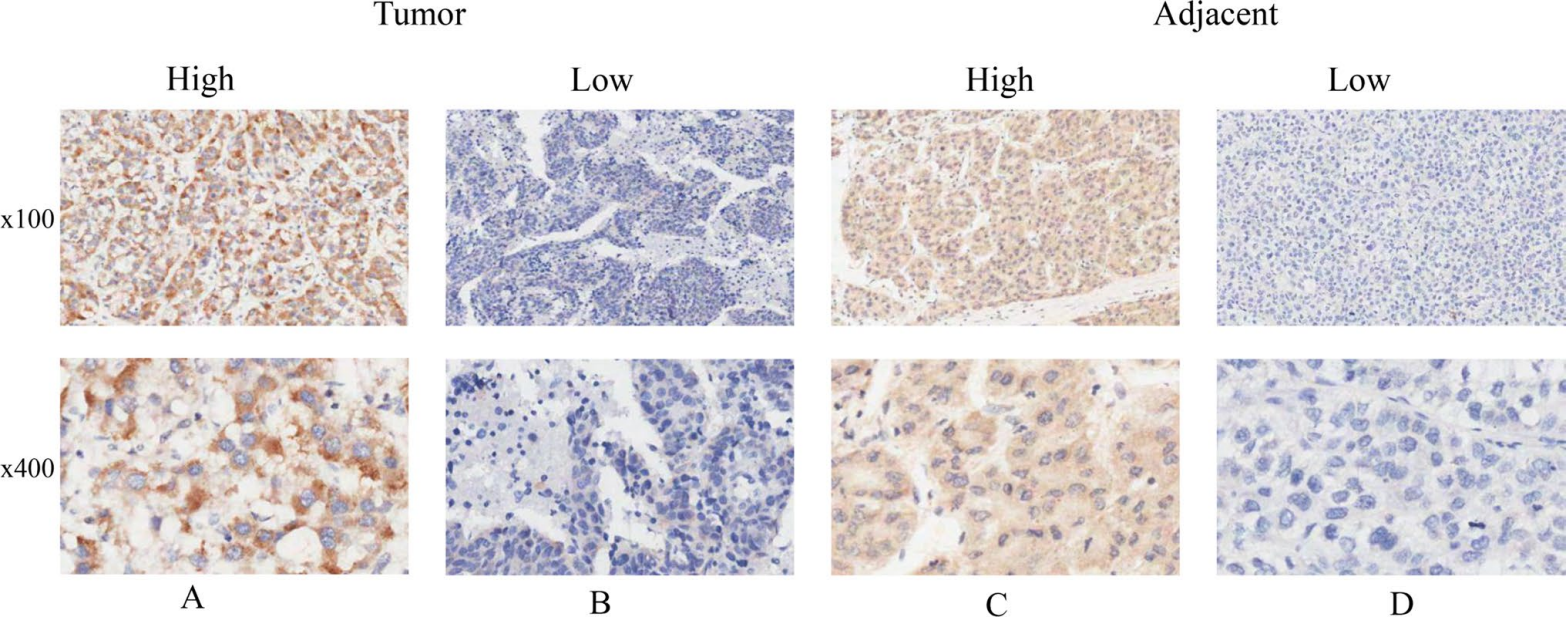
Immunhistochemical analyses of TRIP13 protein levels in HCC and parancancerous tissues. **A** High levels of TRIP13 protein in HCC tumor tissues. **B** Low levels of TRIP13 protein in HCC tumor tissues. **C** High levels of TRIP13 protein in paratumor tissue. **D** Low levels of TRIP13 protein in paratumor tissue

**Fig. 5:**
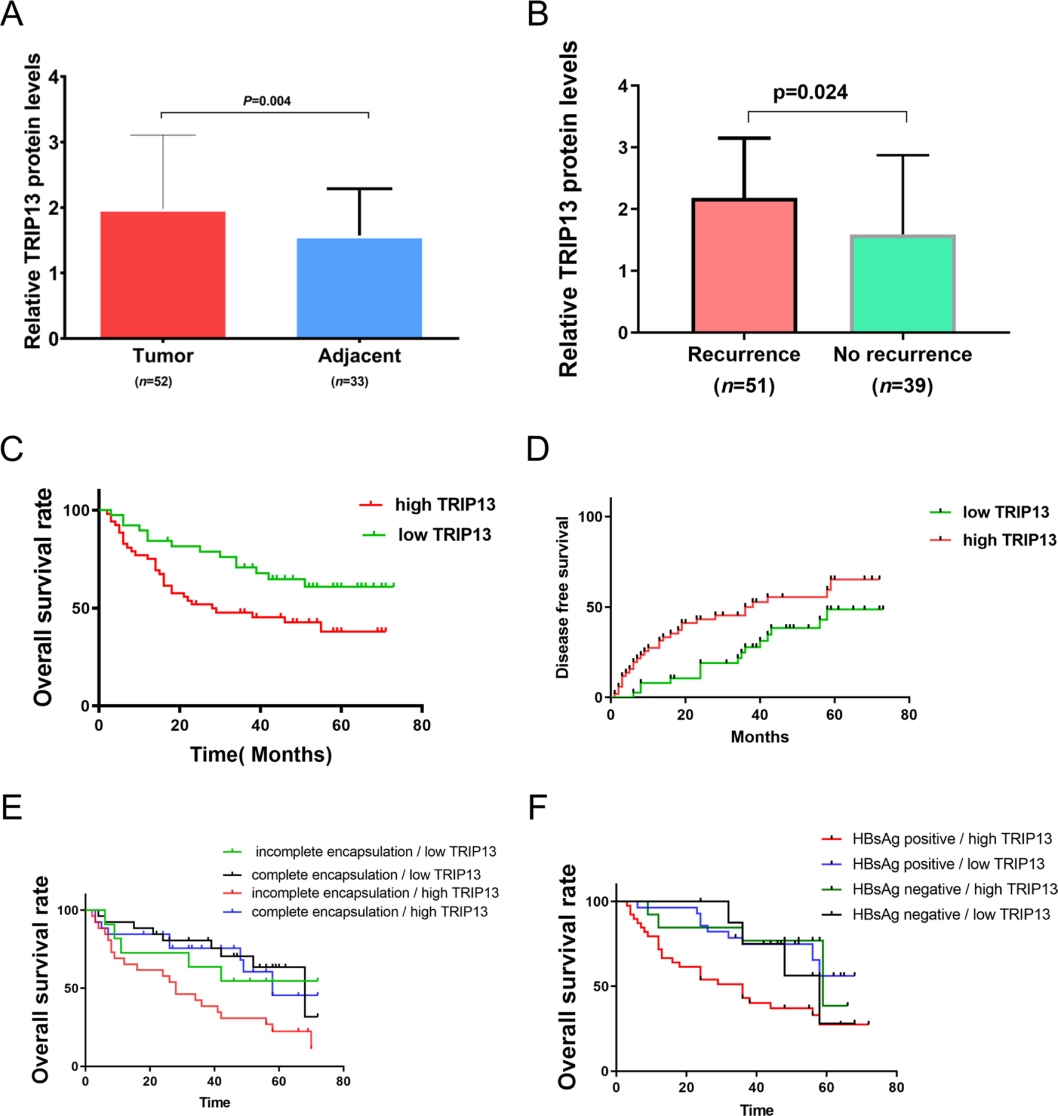
The expression of TRIP13 in HCC and its prognostic significance. **A** The levels of TRIP13 expression in HCC tumor tissues are much higher than paracancerous tissues (P = 0.004) (TRIP13 protein expression levels are expressed as mean ± standard deviation). **B** The protein levels of TRIP13 in HCC are strongly linked to the disease’s recurrence (P = 0.024). **C** Analysis of the Kaplan-Meier method’s connection between the TRIP13 protein levels and patients’ OS (P = 0.025). **D** A Kaplan–Meier analysis of the link between the TRIP13 protein levels and the patients’ disease free survival (DFS) (P = 0.036). **E** Examining the association between overall survival (OS) and the levels of the TRIP13 protein in patients with imperfect encapsulation and complete encapsulation. **F** Correlation between the OS of patients with positive and negative HBsAg and the TRIP13 protein levels

Subsequently, we validated the TRIP13 expression in HCC using an independent cohort of tumor and paratumor tissue samples obtained from our institution (Table 1). Immunohistochemistry was employed to quantify the TRIP13 protein levels in 90 cases of liver and paratumor tissues (Fig. 4). The results showed that 52 cases (57.78%) of tumor tissue and 33 cases (42.22%) of paratumor tissue exhibited elevated levels of TRIP13 protein. The t-test analysis revealed that the immunohistochemistry score of the tumor tissue (1.983 ± 0.118) was significantly higher than the paratumor tissue (1.573 ± 0.075) (*P* = 0.004) (Fig. 5A). Moreover, we found that the recurrence patients had a higher TRIP13 protein level than the nonrecurrence (*P* = 0.024) (Fig. 5B).

Based on the semi-quantitative scoring results of TRIP13 protein via immunohistochemistry in HCC, we investigated the association between high and low levels of TRIP13 protein and clinicopathological parameters of patients. Our analysis showed that TRIP13 protein levels were significantly correlated with the individual’s alpha-fetoprotein (AFP) level, tumor number, and incomplete encapsulation (all *P* < 0.05) (Table 1).

### Validation of prognostic significance of TRIP13 in HCC using an independent cohort

The average survival rate of 90 patients was 49.48% over 5 years, and about 56.67% of the patient experienced recurrence during the follow-up period. Then, we conducted Kaplan–Meier analyses on various groups based on TRIP13 protein levels, and subsequently plotted the corresponding survival curves. Ppatients with high levels of TRIP13 protein had significantly lower chances of surviving for 5 years compared to those with low levels (38.3% vs. 60.9%; *P* = 0.025). However, patients with high levels of TRIP13 protein exhibited better 5-year disease-free survival (DFS) rates than those with low levels of the protein (67.3% vs. 48.4%; *P* = 0.036) (Fig. 5C, D).

The patient cohort was stratified into subgroups to allow for a more detailed examination of the correlation between TRIP13 protein levels and HCC prognosis. Patients with incomplete encapsulation and high TRIP13 protein levels (n = 29) had a shorter OS than those with low levels of TRIP13 protein (n = 11) (*P* = 0.017) (Fig. 5E). In addition, HBsAg-positive patients (n = 71) with elevated TRIP13 protein levels (n = 42) had a shorter OS (*P* = 0.006) (Fig. 5F). Moreover, no significant association was observed between TRIP13 protein level subgroups and OS in patients with complete encapsulation (n = 50) or HBsAg negativity (n = 19) (*P* = 0.587 and *P* = 0.261, respectively).

43.3% of HCC patients had survived without experiencing relapse, while HCC was responsible for 47.78% of all deaths at the end of the follow-up period. According to univariate analyses, the TRIP13 protein levels, TNM stage, and AFP levels (50 g/L vs. >50 g/L) were significantly associated with OS (*P* < 0.05). The levels of TRIP13 protein and AFP were identified as independent predictors of long-term survival in HCC (Table 2). And DFS in HCC patients was affected by the levels of TRIP13 protein, tumor size, number of tumors, TNM stage, and AFP levels (50 g/L versus > 50 g/L) (*P* < 0.05). Moreover, both AFP and TRIP13 protein levels were independent predictors of DFS in HCC (Table 3).

**Table 2:** Univariate and multivariate analysis of prognostic factors of OS.

Factors	Univariate analysis		Multivariate analysis	
	HR (95% CI)	*P* value	HR (95% CI)	*P* value
TRIP13 (high vs. low)	2.021 (1.119–3.651)	0.025	2.315 (1.297–4.362)	0.008
AFP (ng/mL) (≤ 50 vs. > 50)	4.635 (2.131–10.079)	0.009	4.056 (1.691,9.730)	0.001
TNM (I vs. II)	1.955 (1.521–2.513)	0.012	1.910 (1.429–2.554)	0.036
Gender (male vs. female)	0.658 (0.337–1.283)	0.219		n.a.
Age, years (≤ 60 vs. > 60)	0.802 (0.412–1.562)	0.516		n.a.
Tumor number (solitary vs. multiple)	2.501 (0.977–6.401)	0.0559		n.a.
Tumor size (cm) (≤ 5 vs. > 5)	1.542 (0.671–2.315)	0.062		n.a.
Tumor differentiation (I–II vs. III–IV)	1.382 (0.334–5.732)	0.656		n.a.
Encapsulation (no vs. yes)	1.923 (0.854–4.332)	0.115		n.a.
HBsAg (negative vs. positive)	1.033 (0.496–2.155)	0.930		n.a.

**Table 3:** Univariate and multivariate analysis of prognostic factors of DFS.

Factors	Univariate analysis		Multivariate analysis	
	HR (95% CI)	*P* value	HR (95% CI)	*P* value
TRIP13 (high vs. low)	1.913 (1.059–3.455)	0.036	2.361(1.284–3.945)	0.016
TNM (I vs. II)	1.467 (0.814–2.643)	0.021	1.907(1.409–2.582)	0.024
AFP (ng/mL) (≤ 40 vs. > 40)	1.872 (1.235–3.619)	0.033	4.012(1.718–9.371)	0.004
Gender (male vs. female)	0.669 (0.3436–1.304)	0.237		n.a.
Age, years (≤ 60 vs. > 60)	0.869 (0.446–1.692)	0.679		n.a.
Tumor size (cm) (≤ 5 vs. > 5)	2.422 (1.315–4.460)	0.005		n.a.
Tumor number (solitary vs. multiple)	2.716 (1.055–6.995)	0.038		n.a.
Encapsulation (no vs. yes)	1.730 (0.768–3.899)	0.186		n.a.
Tumor differentiation (I–II vs. III–IV)	1.338 (0.322–5.513)	0.693		n.a.
HBsAg (negative vs. positive)	0.979 (0.469–2.042)	0.954		n.a.

### Infiltration of immune cells and TRIP13 expression levels

**Fig. 6:**
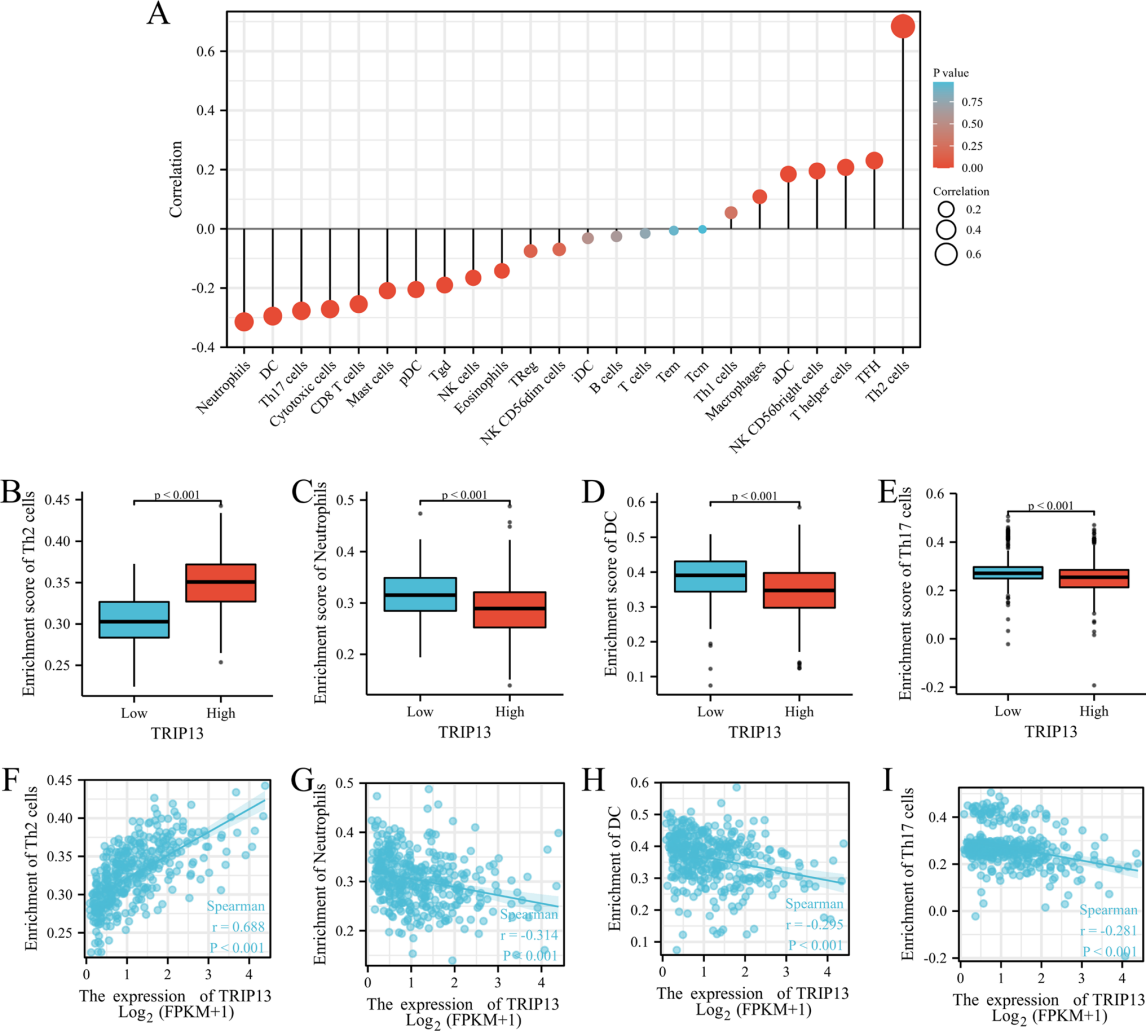
Infiltration of immune cells and TRIP13 expression levels. **A** Identifying the relationship between TRIP13 expression and 24 immune cells’ abundances. **B**–**E** Comparing groups with high and low expression of TRIP13 in terms of immune infiltration levels. **F**–**I** TRIP13 expression and immune cell enrichment fraction

Based on the results of the TCGA cohort analysis, there was a significant correlation between TRIP13 expression levels and the number of immune cell infiltrations (Fig. 6A). The correlation between TRIP13 expression and Th2 cell infiltration was significant (r = 0.688, *P *< 0.001) (Fig. 6B). There was a negative correlation between the levels of TRIP13 expression and the number of immune cell infiltrations of neutrophils (r = − 0.314, P < 0.001), dendritic cells (DC) (r = − 0.295, *P *< 0.001), and Th17 cells (r = − 0.281, *P *< 0.001) (Fig. 6C–E). TRIP13 high expression groups had significantly higher enrichment scores for Th2 cell infiltration than TRIP13 low expression groups, whereas TRIP13 high expression groups had significantly lower enrichment scores for neutrophil, dendritic cell, and Th17 cell infiltration.

## Discussion

Numerous studies have identified various genes and molecules that could serve as diagnostic and therapeutic biomarkers for HCC [17]. Despite multiple factors contributing to the pathogenesis of HCC, patients with advanced disease still exhibit poor prognoses [18]. Our investigation of TRIP13’s association with the prognostic value of HCC in the TCGA and GSE62232 databases, as well as our institution’s independent cohort, confirmed our results and aided in determining TRIP13’s function in HCC patients. Our hypothesis is that the presence of TRIP13 may indicate a potential biomarker for HCC development. While alpha-fetoprotein is a commonly used clinical diagnostic and prognostic predictive marker for HCC, we discovered a negative correlation between alpha-fetoprotein levels and TRIP13 protein levels.

In 2014, Banerjee and colleagues conducted a study examining the impact of TRIP13 on head and neck cancer development [9]. Since then, further research has highlighted the crucial role of TRIP13 in regulating tumor cell survival, expansion, and invasion across various cancer types, including colon, prostate, and breast cancer [9, 18, 19]. The TRIP13 protein, comprising 432 amino acids, is closely linked to chromosomal instability in human cancer and is located in the 5p15 region of chromosome, that makes an essential function in the development and recombination of cellular meiotic DNA breaks, checkpoint signaling, and chromosome synapsis [20]. Reduced levels of TRIP13 protein may cause genomic instability by delaying mitotic arrest, while overexpression of the protein may lead to unexpected interruptions in cell cycle [21].

Numerous studies have reported that TRIP13 protein is overexpressed in various types of human cancer, significantly contributing to tumor cell growth, invasion, and metastasis [9]. Additionally, in a study investigating the protein–protein interactions of TRIP13 in head and neck cancer, suggesting a potential role in promoting tumor drug resistance via the nonhomologous end-joining (NHEJ) signaling pathway. To further understand the association between TRIP13 protein levels and tumor development, we utilized the TCGA and GSE62232 databases to compare TRIP13 protein levels in HCC liver tissue with normal liver tissue. Our IHC results revealed a significantly higher rate of positive TRIP13 expression in tumor tissue, supporting previous findings.

The TRIP13 protein has been extensively studied in animal models, particularly in liver cancer [22]. In a study using a mouse model of HCC with diethylnitrosamine injection, researchers found that the downregulation of TRIP13 protein significantly delayed liver regeneration after hepatectomy, demonstrating the critical role of TRIP13 in hepatocyte repair [23]. Similar results were obtained in studies using mouse HCC cells, where TRIP13 was found to play an essential role in hepatocyte growth and division [9]. Although the precise mechanisms are still unclear, the study suggested that the EGFR-mTOR signaling axis may be involved in the oncogenic function of TRIP13. While the exact physiological function of TRIP13 remains unknown, it has been implicated in various aspects of HCC development.

Recent studies have demonstrated the vital role of immunologic cell infiltration in tumor microenvironment in regulating the progression, metastasis, recurrence, and response to immunotherapy of hepatocellular carcinomas [24]. Immune cell infiltration has a substantial impact on the immune microenvironment, tumor development, treatment strategies, and the likelihood of relapse [25]. Investigating immune cell infiltration in tumors provides valuable insights into the efficacy and mechanisms of immunotherapy for liver cancer, which is a promising treatment option for HCC [26].

This investigation aimed to assess the prognostic value of TRIP13 in HCC and its impact on immune cell infiltration, thus providing a theoretical basis for using TRIP13 as a prognostic target. Nonetheless, this study has certain limitations. Firstly, retrospective studies are inherently biased due to their nature. Secondly, the size of our validation sample was small, which hindered the comparison of patients by TNM stage for overall survival (OS). Moreover, the evaluation of immune cell infiltration was based solely on a public database, and further validation through in vivo and in vitro studies is warranted.

## Conclusion

This study discovered that TRIP13 is upregulated in HCC and is associated with clinicopathological characteristics and immune cell infiltration in HCC patients. In addition, high TRIP13 expression was associated with a poor prognosis in HCC patients, suggesting that it could be used to predict the prognosis of HCC patients.

### Supplementary Information

Below is the link to the electronic supplementary material.

Supplementary file 1 (DOCX 22 KB)

## Data Availability

Data is available at TCGA and NCBI GEO (accession numbers: GSE62232). Our own cohort data can be obtained by contacting the corresponding author.

## Abbreviations

TRIP13: Thyroid hormone receptor interactor 13
HCC: Hepatocellular carcinoma
TCGA: The Cancer Genome Atlas
GEO: The Gene Expression Omnibus
OS: Overall survival
DFS: Disease-free survival
DSS: Disease specific survival
PFI: Progress free interval
AFP: Alpha-fetoprotein
HBsAg: Hepatitis B surface antigen
HR: Hazard ratio
CI: Confidence interval
