# Supplementary material for: TRIP13 overexpression in hepatocellular carcinoma: implications for poor prognosis and immune cell infiltration
Source: Discov Oncol. 2023 Sep 22;14:176. doi: 10.1007/s12672-023-00792-2 (PMC10516817; doi:10.1007/s12672-023-00792-2)
Supplement: Supplementary file 1 — (DOCX 22 KB) [file 12672_2023_792_MOESM1_ESM.docx]

**Supplementary Tables**

**Table S1** Association of TRIP13 levels with clinicopathological parameters of HCC patients from TCGA.

| Parameters | TRIP13 expression | | | P value |
| --- | --- | --- | --- | --- |
|  | Low (n=187) | | High (n=187) |  |
| Gender, n (%) |  |  | | 0.825 |
| Male | 128 (34.2%) | 125 (33.4%) | |  |
| Female | 59 (15.8%) | 62 (16.6%) | |  |
| Age, meidan (IQR) | 63 (52, 69) | 60 (51, 68) | | 0.229 |
| T stage, n (%) |  |  | | **< 0.001** |
| T1 | 114 (30.7%) | 69 (18.6%) | |  |
| T2  T3  T4 | 33 (8.9%)  33 (8.9%)  4 (1.1%) | 62 (16.7%)  47 (12.7%)  9 (2.4%) | |  |
| N stage, n (%)  N0  N1  M stage, n (%)  M0  M1 | 124 (48.1%)  1 (0.4%)  128 (47.1%)  3 (1.1%) | 130 (50.4%)  3 (1.2%)  140 (51.5%)  1 (0.4%) | | 0.623  0.355 |
| AFP (ng/mL), meidan (IQR) | 6 (3, 42) | 35 (7, 1865) | | **< 0.001** |
| Child-Pugh grade, n (%)  A  B&C | 121 (50.2%)  12 (5.0%) | 98 (40.7%)  10 (4.1%) | | 0.902 |
| Vascular invasion, n (%)  Yes  No | 42 (13.2%)  124 (39%) | 68 (21.4%)  84 (26.4%) | | **< 0.001** |
| Tumor status, n (%) |  |  | | **0.002** |
| Tumor free | 116 (32.7%) | 86 (24.2%) | |  |
| With tumor | 61 (17.2%) | 92 (25.9%) | |  |
| OS event, n (%) |  |  | | **0.013** |
| Alive | 134 (35.8%) | 110 (29.4%) | |  |
| Dead | 53 (14.2%) | 77 (20.6%) | |  |
| PFI event, n (%) |  |  | | **0.013** |
| Alive | 108 (28.9%) | 83 (22.2%) | |  |
| Dead | 79 (21.1%) | 104 (27.8%) | |  |

| **Table S2** Univariate and multivariate analysis of prognostic factors of OS from TCGA. | | | | | |
| --- | --- | --- | --- | --- | --- |
| Factors | Univariate analysis | |  | Multivariate analysis | |
|  | HR (95% CI) | *P*  value |  | HR (95% CI) | *P* value |
| TRIP13 (high vs. low) | 1.927 (1.353-2.742) | **<0.001** |  | 2.023 (1.282-3.193) | **0.002** |
| T stage (T1&T2 vs. T3&T4) | 2.598 (1.826-3.697) | **<0.001** |  | 2.581 (1.661-4.009) | **<0.001** |
| M stage (M0 vs. M1) | 4.077 (1.281-12.973) | **0.017** |  | 3.238 (0.948-11.058) | 0.061 |
| Gender (male vs. female) | 1.261 (0.885-1.796) | 0.200 |  | - | n.a. |
| Age, years (≤ 60 vs. > 60) | 1.205 (0.850-1.708) | 0.295 |  | - | n.a. |
| N stage (N0 vs. N1) | 2.029 (0.497-8.281) | 0.324 |  | - | n.a. |
| Histologic grade (G1&G2 vs. G3&G4) | 1.091 (0.761-1.564) | 0.636 |  | - | n.a. |
| Child-Pugh grade (A vs. B&C) | 1.643 (0.811-3.330) | 0.168 |  | - | n.a. |
| Vascular invasion (no vs. yes) | 1.344 (0.887-2.035) | 0.163 |  | - | n.a. |

| **Table S3** Univariate and multivariate analysis of prognostic factors of DSS from TCGA. | | | | | |
| --- | --- | --- | --- | --- | --- |
| Factors | Univariate analysis | |  | Multivariate analysis | |
|  | HR (95% CI) | *P* value |  | HR (95% CI) | *P* value |
| TRIP13 (high vs. low) | 2.383 (1.501-3.783) | **<0.001** |  | 6.376 (2.325-17.486) | **<0.001** |
| T stage (T1&T2 vs. T3&T4) | 3.639 (2.328-5.688) | **<0.001** |  | 2.714 (1.138-6.475) | **0.024** |
| M stage (M0 vs. M1) | 5.166 (1.246-21.430) | **0.024** |  | 14.770 (2.493-87.495) | **0.003** |
| Child-Pugh grade (A vs. B&C) | 2.560 (1.123-5.834) | **0.025** |  | 6.345 (2.110-19.084) | **0.001** |
| Gender (male vs. female) | 1.230 (0.780-1.937) | 0.373 |  | - | n.a. |
| Age, years (≤ 60 vs. > 60) | 0.846 (0.543-1.317) | 0.458 |  | - | n.a. |
| N stage (N0 vs. N1) | 3.612 (0.870-14.991) | 0.077 |  | - | n.a. |
| Histologic grade (G1&G2 vs. G3&G4) | 1.086 (0.683-1.728) | 0.726 |  | - | n.a. |
| Vascular invasion (no vs. yes) | 1.277 (0.707-2.306) | 0.418 |  | **-** | n.a. |
